# 薄层色谱与质谱联用的研究进展

**DOI:** 10.3724/SP.J.1123.2022.03038

**Published:** 2023-01-08

**Authors:** Xiaowei ZOU, Xing LIU, Jianming ZHANG

**Affiliations:** 上海科哲生化科技有限公司, 上海 201108; Shanghai Kezhe Biochemical Technology Co. Ltd., Shanghai 201108, China

**Keywords:** 薄层色谱, 质谱, 接口, 综述, thin-layer chromatography (TLC), mass spectrometry (MS), interface, review

## Abstract

薄层色谱(TLC)是一类非常实用的液相色谱方法,由于其装置简单、操作便捷、灵活、通量高、成本低,以及样品前处理简单等优点,在许多行业的检测中都有广泛的应用并扮演着重要的角色。随着现代检测技术的不断发展以及各种检测技术综合应用程度的加深,薄层色谱与质谱的联用(TLC-MS)也成为这一方法的重要发展方向。随着我国医药、食品、科学仪器等事业的不断发展和升级,相信薄层色谱-质谱联用技术可以起到更好的作用,并迎来发展的契机。该综述将目前薄层色谱-质谱的联用形式分为3类,一是接口仪器的间接联用,二是质谱对薄层板的原位检测,三是质谱对薄层分离过程的实时监测,并按此分类对典型的联用形式进行了总结和简要描述。随着薄层色谱-生物自显影技术的广泛使用,薄层色谱与质谱联用的技术方法极大地提高了食品、药用生物活性物质的研发效率。目前,薄层色谱与质谱联用发展的主要瓶颈是“即插即用”型部件的设计和商品化。具有实时监测功能,同时又兼备灵活扫描功能和高通量特点的TLC-MS技术也很令人期待。此外,不同种类TLC-MS解吸-电离技术的对比研究也是有待讨论的应用问题。

## Abstract (en)

Thin layer chromatography (TLC) is a very useful liquid chromatography approach. The simple device, convenient operation, versatility, high throughput capabilities, low cost, and simple sample pretreatments make it widely employed in various fields. In recent years, TLC-MS has become one of the most prominent trends for this technology as developments of modern analytical technology and comprehensive application of different approaches. With the development and upgrading of medicine, food, and scientific instrument industries, it is believed that TLC-MS technology should play a better role and obtain an opportunity for development. This study reviewed TLC-MS interface technologies (most of which are in recent 10 years) based on more than 150 studies and classified these TLC-MS technologies as three strategies. The first is indirect coupling using commercially available interface instruments. The second is TLC-in-site detection directly with special MS ion source devices like fast-atom-bombardment desorption ionization, matrix-assisted laser desorption ionization, surface-assisted laser desorption ionization, electrospray-assisted laser desorption ionization, laser-induced acoustic desorption/electrospray ionization, electrostatic-spray ionization, easy ambient sonic-spray ionization, desorption sonic spray ionization, ionization using “desorption/ionization resource”, ionization using “molecular ionization-desorption analysis source”, multiwavelength laser desorption ionization, ionization using flowing afterglow-atmospheric pressure glow discharge, ionization low-temperature plasma probe, desorption/ionization induced using neutral clusters, ionization using inductively coupled plasma and so on. These MS analyses are performed after TLC development, thus, the relative position of the chromatographic bands on TLCs is invariable, and this analysis can be regarded as static detection, though flexible travel stages or conveyor belts can be introduced to move TLC plates. The third strategy is to monitor TLC run using MS in real-time just as the monitor employed in HPLC, in which the chromatographic bands are still moving. This strategy is generally run on forced-flow TLC techniques and is less examined. The typical coupling technologies (especially appeared in recent ten years) are summarized and briefly described in this study. TLC-MS has greatly enhanced the research efficiency of bioactive substances for food and drugs due to the widespread usage of TLC-bioautography technology. Nowadays, the main bottleneck in the development of TLC-MS is the design and commercialization of “plug and play” components. The high-throughput and real-time monitoring TLC-MS technology with flexible scanning functions is also expected. Furthermore, the comparative studies of different kinds of desorbing-ionizing technologies are also application problems for further discussion.

薄层液相色谱(TLC)是一种常见且有效的液相色谱分离技术,在食品^[1⇓-3]^、医药^[4⇓-6]^、化妆品^[7]^、刑侦与消防^[8]^、工业工艺^[9,10]^和环境检测^[11,12]^等诸多领域都有广泛应用。尤其在医药领域,TLC几乎是所有化学药工艺研发过程中必不可少的中控方法,同时TLC在中药和药物辅料的检测中也占有非常重要的地位。据统计,2020版《中国药典》第一部中药分册中,有近94%的品种使用了TLC作为质量控制分析方法;2020版《中国药典》第四部辅料分册中有接近40个品种使用了TLC。

尽管柱液相色谱发展迅速,但TLC成本低、操作灵活便捷、样品前处理简单、通量高、仪器联用便捷等优势仍是柱色谱难以达到的。近年来TLC技术有两个发展趋势,一是检测手段多元化,例如与多种检测器联用(如红外光谱^[13,14]^、拉曼光谱^[3,15]^、表面增强拉曼光谱^[16,17]^、质谱(MS)^[18]^、离子迁移谱^[19]^、荧光光谱^[20]^、气相色谱^[21]^、液相色谱^[22]^、核磁共振^[23]^等),或者与生物技术相结合从而形成效应导向分析(生物自显影)^[24,25]^;二是检测灵活化和便捷化,例如通过3D打印或平面打印等方法,薄层色谱正逐步成为一种“办公室色谱”技术^[26⇓⇓-29]^,而手机终端上的数据处理也使数据分析更加简便高效^[30]^。本文综述的薄层色谱-质谱联用(TLC-MS)技术是多元化检测趋势的代表,当前TLC仪器联用技术中最为活跃的研究方向之一^[18]^,尤其在食品^[31⇓⇓-34]^和医药领域(如化学药^[35]^、天然产物或植物药^[32,36⇓⇓-39]^、临床检测^[35,40⇓-42]^等方面)有广泛的应用空间。

目前TLC-MS联用的形式大致可以归纳为3种。

第一种是通过独立的接口仪器装置将薄层上的谱带转移出来,再送入质谱进行分析。

第二种是直接在薄层板上进行的“原位”质谱分析,即在薄层展开完成之后再进行质谱检测,此时谱带在薄层板上的相对位置已经固定,质谱扫描时先固定好质谱电离源的位置和角度,然后对正薄层板的位置再进行扫描检测。这种检测方式可称为静态检测;尽管也可以通过传送带等方式移动薄层板,使固定相上的谱带依次通过质谱扫描检测区域而得到连续的色谱图,但由于谱带上的相对位置并未发生变化,因此仍应归属为静态检测。

第三种是在薄层板上直接进行实时监测,这种监测方式与高效液相色谱(HPLC)相似,在谱带展开的同时进行检测,只要流动相带着样品成分经过检测点,检测器就会对相应的谱带有信号响应。检测时谱带在薄层板上的相对位置仍处于变化中,因此可称这种检测为动态检测。

## 1 TLC-MS接口仪器装置

对于上文所述的第一种TLC-MS检测方法,其目标是将薄层上的谱带提取出来,再送入质谱进行分析。常见的转移操作有两种,一种是手动操作,即在薄层分离后进行刮板操作,再用有效的提取液从吸附剂中提取目标化学成分,进行纯化和浓缩处理后进行质谱分析,也称为间接上样或者离线联用;另一种则是借助TLC-MS接口仪器用提取液直接对目标谱带进行提取,自动过滤后送往质谱电离源。

对于自动化的TLC-MS接口仪器来说,使用过程主要包括3个操作步骤,即TLC分离、目标成分的接口转移、质谱分析^[43]^。商业化仪器接口装置生产商主要有瑞士CAMAG、美国Advion等,目前我公司也开发了TLC-MS接口的商品化仪器,原理上与进口仪器相同,结构上与之相似(如图1所示)。基于接口仪器装置的TLC-MS联用检测,在化学合成领域、食品、生物医药、环境检测方面都取得了不错的应用效果,既可用于有机成分的分析也可用于元素分析^[44⇓-46]^。

**图1:**
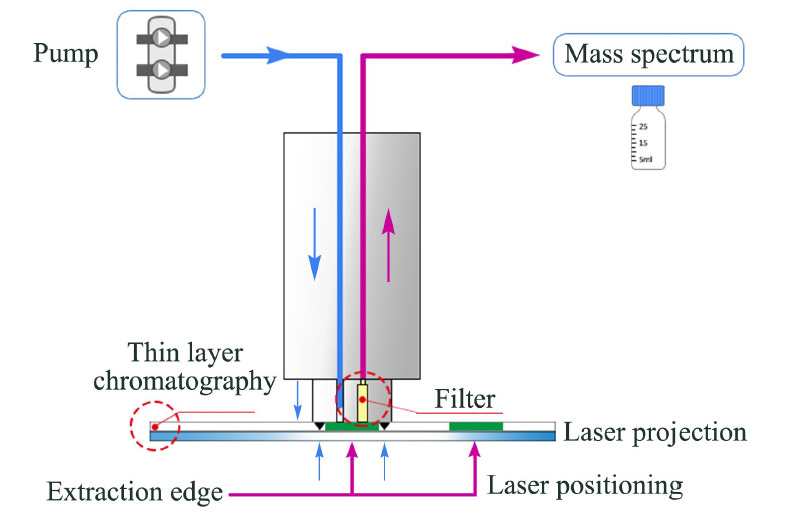
薄层色谱-质谱接口仪器原理

使用过程中,接口仪器对准TLC板上的目标谱带后,与之紧密接触,然后使用指定体积的溶剂(如甲醇等)对它进行洗脱,过滤后通过内部管路输送至质谱进行分析,最后对接口仪器管路进行反向冲洗。这种设计不易引入不溶性杂质,因此也无需再在MS前加预柱^[40,47⇓-49]^。但是常见的问题是取样探头和薄层之间的密封不足,时常会有少量洗脱液析出,造成谱带的扩散,应用超薄薄层色谱(UTLC,固定相厚度一般为1~50 μm)时可取洁净滤纸放于取样探头和薄层之间以缓解这一问题^[50]^。

Morlock研究组^[49]^使用飞行时间质谱考察了TLC-MS接口仪器方法的定量性能,实验结果表明6种染料检测的平均精密度优于10%,平均相关系数为0.9975。之后他们将生物自显影薄层技术与轨道离子阱质谱相结合,在同一高效薄层色谱(HPTLC)板上对样品成分进行活性筛选,随后用制备薄层色谱与氢核磁共振联用进行了制备和表征,全面了解了活性成分的属性^[23]^;他们还使用由四极杆质谱组成的HPTLC-MS系统测定了葡萄酒中甘油、葡萄糖酸、氨基酸和糖含量,并建立了氨基酸指纹图谱^[51]^。

## 2 TLC-MS原位检测

TLC-MS技术联用的第二种形式则是在薄层板上直接进行原位检测,这种方法的样品前处理相对简单,常用于低聚糖、氨基酸、脂类等基质成分复杂的生物成分分析。这种仪器技术的关键在于兼容模式电离源的设计,目前报道的原位检测电离源主要有快原子轰击电离源,激光解吸电离源(LDI)、电喷雾电离源、大气压化学电离源、大气压余辉电离源和等离子体类电离源。样品成分由于吸附在薄层板上,因此需要解吸和电离,常见的解吸方式主要有加热解吸、快原子轰击或高能Ar离子解吸、激光和喷雾辅助解吸。

### 2.1 薄层色谱与快原子/快离子轰击源质谱的联用

快原子轰击电离源质谱(FAB)属于二次离子化质谱(LSIMS),即通过高能量的一次离子束轰击样品表面,使样品表面的原子或原子团吸收能量而从表面溅射产生二次粒子,并对这些带电粒子进行质谱分析。FAB一般适用于极性小分子化合物(<2000 Da),在一定真空条件下使用加速原子轰击(一般为Ar原子)样品成分,使之电离并进入质谱。与TLC联用时,可将分离后的薄层进行切割,再置入真空室进行解吸、电离和检测。操作时一般会在目标谱带上添加间硝基苯甲醇、三乙醇胺或甘油、硫代甘油等黏性基质的甲醇溶液,起到能量传递、清洁表面、提供电荷以及延长信号持续时间的作用。与快原子轰击类似的还有离子轰击(如5~20 kV加速的Cs^+^离子)^[52]^。这类方法在检测中常有谱带扩散问题,重复性稍差,而且快速原子或离子轰击TLC板表面时产生的凝胶颗粒也可能对真空系统(如涡轮分子泵)有损害。对于谱带扩散的问题,可以通过原位浓缩样品后再检测,效果会有所改善^[53]^。胡耀铭等^[54]^将纸薄层和薄层板分别与双聚焦质谱联用,对氨基酸、绞蓝皂苷降解化合物进行了分析。近年来这种技术在TLC-MS原位检测的应用中并不多见。

### 2.2 薄层色谱与激光辅助解吸/烧蚀源质谱的联用

激光在烧蚀、解吸或蒸发固体样品方面是十分有力的工具。通常只需使用脉冲激光束就可以从样品中解吸(或烧蚀)并电离部分质量的样品成分^[55]^,具有样品前处理简单、耗材少、分析周期短、功率密度高和空间分辨率出色等优点,能够在小范围内进行采样和电离,既可以进行烧蚀以支持元素分析、同位素分析和分类分析,也可以用于解吸有机成分进行后续分析。这种技术已经广泛应用于与质谱的联用,常被称为激光解吸/烧蚀电离(LD/LA)^[56]^。

LD/LA技术中最常见的应属基质辅助激光解吸电离(MALDI),它一般适用于极性化合物的解吸和电离,在灵敏度和通用性方面,MALDI常优于FAB。与MALDI相似的技术还有表面辅助激光解吸电离(SALDI), MALDI在相对分子质量较低的区域(如*m/z*<700)基质分子干扰较明显,相对来说使用SALDI技术时这一区域影响较小。MALDI在使用中(包括与TLC联用时)需要*α*-氰基-4-羟基肉桂酸、2,5-二羟基苯甲酸、芥子酸等基质的辅助,用于吸收激光的能量并能转移质子。对于MALDI来说,基质类型、分析物与基质的比率、基质应用的技术方法、结晶过程、萃取溶剂、萃取时间、激光枪及其位置等因素都会影响MALDI中的信号强度^[57]^。激光照射条件对于MALDI或者SALDI技术十分重要,一般来说TLC-MALDI-MS联用时脉冲激光的效果要优于连续激光^[58]^。

TLC与MALDI表面显然有区别,对此有两种处理方法。一种方法是将TLC板上的成分转移出来,进行离线TLC-MALDI-MS分析,如以下几个例子:

(1)通过薄层层析的方式将TLC上的成分迁移到MALDI基质板上。Mehl等^[59]^在TLC分离结束后,将TLC板和MALDI基质板并排平行放置于同一平面上,沿着与TLC板展开垂直的方向进行二次谱带洗脱,使谱带从TLC板迁移至MALDI基质板上。

(2)首先在不锈钢板上制备基质层并干燥;将提取液喷涂在已经完成分离的TLC板上,并将以上两块板相向重叠并压合在一起,在压力作用下TLC中成分扩散转移到基质层中,之后移除不锈钢板,对TLC进行质谱分析^[60]^。此外还可以通过印迹技术进行物质转移,如Goto-Inoue等^[61]^在TLC展开后,将斑点从该薄层转移到聚偏二氟乙烯(PVDF)膜上,再将PVDF膜附着到MALDI靶板上进行成像分析;这种方法的优点是一定程度上可以减弱分析物在TLC板上的扩散,但是不足之处是提取效率不够高,部分样品成分有可能进入TLC固定相颗粒的内孔而不易提取,无法得到令人满意的回收率。

(3)通过刮板等方法将目标成分从薄层板上转移下来,再进行后续的MALID检测,如Manikandan等^[62]^使用TLC从培养的微生物中分离盐生类胡萝卜素,通过刮板取样,使用液液微萃取法处理后进行MALDI-TOF分析。

另一种方法是通过溶剂挥发-基质沉降或浸涂等方式直接在薄层板上构建基质,使用这种方法后可对TLC进行原位检测^[63⇓⇓-66]^。通常选择铝箔(或其他金属)材料作为TLC板基底,一方面便于向样品成分传输电子,另一方面也可以为离子飞向质谱入口提供加速电压^[67]^。如果使用玻璃基板的TLC,尽管玻璃材质不会妨碍加速电压的提供,但显然会影响电子的传输功能,因此所需的激光光强也会更大。对于这种直接构建基质的方法来说,它的不足之处是有样品扩散的问题,而造成这一问题的主要原因之一是结晶过程引起的表面张力变化。

MALDI技术可分为真空上样和大气压上样。当前与TLC联用的应用中,仍以真空上样居多,操作时常用双面胶带将TLC板连接到MALDI源相关的探针装置上进行分析。而大气压上样操作时可以将基质涂覆于薄层板上,之后进行脉冲激光的解吸和电离,如Salo等^[68,69]^将基质(*α*-氰基-4-羟基肉桂酸)喷涂于TLC板的成分谱带上进行小分子分析。

TLC固定相的厚度和粒径大小会对质谱信号有影响,一般认为固定相的厚度对质谱信号的影响比薄层固定相粒径大小更明显,而较薄固定相可以获得更优的信噪比^[70]^。因此不难理解UTLC-MALDI-MS在灵敏度上的表现更为出色,不少报道也证实了这一点^[68,71,72]^。Kurganov等^[73]^综述了整体固定相UTLC与MALDI联用的情况,通常无机整体固定相基质在小质量区域内易产生干扰信号,此时可以调整分析组分与基质的物质的量之比,或使用一些特殊基质来优化。相对来说,有机聚合物构成的整体固定相更利于避免类似问题,例如由聚合物纳米纤维(聚丙烯腈、聚乙烯醇和碳纳米纤维)制得的玻璃碳超薄层等分离材料都可用于MALDI-MS检测。Mernie等^[74]^则是使用了含有离子液体的纳米材料作为基质进行样品的辅助电离。此外,质谱检测器的引入也使得一些特殊的材料可以用作TLC的固定相,例如有色的碳纳米纤维固定相,由于它本身有颜色而且易引起荧光淬灭,因此这种材料在TLC技术中的应用受到了限制,但如果与MS联用就可以避免这个问题。

尽管UTLC在质谱的联用中有信噪比的优势,但选择固定相时还需要兼顾上样容量等因素。例如Griesinger等^[70]^研究考察了200、100和60 μm硅胶TLC板上分离磷脂酰胆碱样品的情况,60 μm厚度的薄层具有最好的信噪比,但出于上样量、分离能力,以及检出限等因素的综合考虑,他们认为100 μm的HPTLC板是较优的选择。

除MALDI外,SALDI是另一种重要的LDI技术,可用于多种生物有机小分子的检测,如肽、聚糖、固醇类化合物等。在操作中常使用少量提取液将待测成分提取至液滴里,再加上辅助材料(即基质,如碳粉末)悬浮于液滴上,然后结合激光解吸电离。它的原理与MALDI相似,基质材料可吸收激光能量并再分配,之后对目标成分进行解吸和电离,这个过程存在着多种机制,如热解吸、电荷转移、纳米结构引发机制、相转变机制、表面重组、表面等离子体等,一般来说热解吸更被广泛接受。常见的基质材料主要有碳材料(活性炭、石墨、富勒烯、石墨烯、纳米管、纳米金刚石、碳量子点等)、硅(如硅纳米颗粒、硅纳米线、硅纳米柱阵列等)、金属(金、银、铂等)和金属氧化物(氧化锌、氧化钛等)材料等无机材料,或者复合材料(量子点、金属-有机骨架、共价-有机骨架等)等^[75⇓⇓⇓⇓⇓⇓⇓-83]^。

Chen等^[84,85]^将TLC与SALDI-MS的方法相结合,使用活性炭作为辅助材料建立了TLC-SALDI-MS方法,进行有机合成反应监测,发现离子表面活性剂(如对甲苯磺酸、十二烷基硫酸钠、烷基三甲基溴化铵)有利于解吸和提高离子化效率^[86]^。表面辅助材料的量值对解吸-电离效率有显著的影响,例如过多的活性炭可能导致目标成分的死吸附,而有时用铅笔在分离后的薄层谱带上涂一下也会有不错的效果^[87]^。Kawasaki^[88]^等在TLC分离之后,用磁控溅射装置在其表面进行金属沉积,形成一层均匀的纳米颗粒层(如铂),之后用表面辅助激光解吸/电离(成像)质谱分析。

总体来说,无论是MALDI还是SALDI与TLC联用时,都有以下几点不足:(1)样品处理和引入真空过程较麻烦。(2)常需要将TLC板切割成小块。(3)检测挥发性或半挥发性化合物时,灵敏度低。(4)定量分析时重现性不理想。(5)吸附剂表面的分析物浓度低,而且基质溶液存在扩散作用,这可能会进一步降低分辨率和局部浓度^[73]^;对于这个问题,可以使用复合基质或无溶剂基质进行缓解^[89,90]^。(6)MALDI电离通常都需要经过质子化、阳离子化或去质子化过程,非极性或低极性化合物难以通过MALDI-MS进行分析;对此,可以使用一些盐对样品成分(如一些醇、酚、胺、甾体等物质)进行衍生,如Esparza等^[91]^提出了一种简单的衍生化方法,可用于含羟基但极性较弱化合物的TLC-MALDI-MS分析,方法是在薄层分离后用3-溴丙酰氯和吡啶对谱带进行衍生化(酰化、季铵化),他们在几种哺乳动物和植物甾醇、酚和萜烯醇上进行了测试,结果表明衍生产物含有永久正电荷。

### 2.3 薄层色谱与ESI源质谱的联用

ESI源是最常见的电离源之一,解吸电喷雾电离(DESI)源结构与之相似,但样品前处理方面要求较低。

本文在第1节所介绍了TLC-MS的接口仪器装置,将这种接口设计与ESI源相结合,可以形成独立的“表面萃取-电喷雾电离源”^[52,92]^。使用时首先将探头对准TLC板上的目标谱带,与之紧密接触,然后使用指定体积的溶剂对它进行洗脱,过滤后送至ESI源进行喷雾电离。Berkel等^[93]^在这类系统的相关研究中发现,在从TLC中提取样品成分的过程中,表面张力的作用影响明显。在具有吸湿性的极性表面上,这种装置的采样效率可能会受限制,为此,Walworth等^[94]^将正相HPTLC板上分离的肽通过印迹法转移到疏水性的RP C8 HPTLC板上,再用TLC-ESI-MS系统进行成分分析。

如前文介绍,激光辅助是质谱分析方法中常借助的技术方法,在激光照射过程中,样品的表面会同时解吸产生中性粒子、分子和分析物离子。然而,离子产率相对较低,即使添加有机基质以辅助电离(如MALDI),分析物离子的产率也远低于中性粒子的产率。因此,常将激光与各种离子化方法结合起来,以提高电离效率^[56]^。这其中就包括了ESI源电离的方式,即电喷雾辅助-激光解吸电离(ELDI),如Lin等^[95]^通过ELDI建立了离子阱质谱和TLC联用的仪器系统,使用激光照射TLC上的样品谱带并用ESI喷雾电离解吸出来的样品成分;他们在实验中验证了正相和反相薄层色谱的分离模式,质谱正离子和负离子检测模式,充分说明了这种联用方式的可行性。

激光解吸和电喷雾相结合的方法除上述类型外,还有另外一种应用称为激光诱导声波解吸/电喷雾电离(LIAD)。在ELDI技术中脉冲激光作用于TLC表面的谱带,与之不同的是,LIAD中脉冲激光主要作用于薄层板背面^[52]^。二者的原理也有差别,ELDI中样品成分通过激光解吸并进入电喷雾后,与电喷雾液滴融合,通过电荷转移作用或随着电喷雾液滴的不断挥发-爆炸作用而带电;而在LIAD中脉冲激光束照射薄层板背面,引起三角形冲击脉冲,并通过基板传播到固定相层样品谱带一侧,目标成分在此作用下以中性分子、溶胶以及微量产物离子的形式进入电喷雾,随后的电离过程与ELDI相似^[56]^。Cheng等^[96]^应用反相C18和正相硅胶薄层,应用TLC-LIAD-MS分析了药物、染料等成分。实验中,为了防止TLC板上的振动、提高能量传递效率,他们在TLC基板的背面贴上了一个玻璃显微镜载玻片,并在玻璃片和TLC基板之间涂上黏性溶液(甘油或聚乙二醇)。由于冲击波需要从TLC的底板(一般为金属基板)传递到固定相表面,因此LIAD-ESI通常需要更高的激光能量,其空间分辨率通常也不及ELDI,近年来报道较少。

上述解吸方法都是作用于某一个谱带进行的解吸、电离,需要手动的方式对正TLC板的位置。为了满足自动化的需求,如今TCL-MS系统常装配有传送带或者可灵活运动的平台装置,用于移动和定位TLC板。如Cheng等^[97]^以此构建了高通量、敞开式的TLC-ELDI-MS分析系统,使用激光对TLC板上的样品进行辅助解吸,电喷雾电离,之后用离子阱或四极杆质谱进行分析。

DESI源也可用于TLC谱带成分的分析^[98⇓-100]^,该电离源以超过100 m/s的速度向TLC板表面喷洒直径<10 mm的带电溶剂液滴,撞击样品表面来进行成分的解吸和电离^[101]^。影响其作用效果的主要因素有分析成分的性质、分析物表面和溶剂系统,以及离子源的参数(如雾化喷头到样品表面的角度和距离、样品到MS入口角度和距离、雾化气体压力和溶剂流速和毛细管电压等)^[102]^。DESI源在UTLC上的解吸-离子化效率一般远高于TLC及HPTLC,迁移距离也更小。Kauppila等^[103]^对比了DESI对多孔二氧化硅薄层、UTLC、聚甲基丙烯酸甲酯和聚四氟乙烯表面样品的解吸电离情况,研究结果表明:(1)这些表面对样品电离不会有抑制效应;(2)在喷口与表面距离以及喷雾流速方面,各种表面材料的要求各不相同;(3)UTLC在实验过程中(实验条件为喷雾流速7 μL/min,距离3~4 mm)表现出了足够的机械强度,而且比HPTLC有更高的解吸-电离效率;(4)加热有助于提高解吸-电离效率,而盐对解吸-电离效率影响较小。值得一提的是喷雾流速过大时,TLC或HPTLC层中松散的二氧化硅颗粒可能通过溶剂喷雾而扩散,给质谱系统带来潜在的污染风险,而UTLC层可以避免这种情况;但是也应注意,流速过大时UTLC固定相层可能因机械强度不足而损坏。

此外,Girault研究小组^[104]^建立了静电喷雾电离(ESTASI)源,这种电离源与ESI相似但不需要使用载气,系统中喷雾端和样品板之间维持着高电压差,利用电容耦合效应,在喷雾端处的溶液表面聚集电荷,当静电斥力大于张力时即发生喷雾电离,朝向带相反电荷的样品板喷射。他们通过ESIASI搭建了HPTLC与线性离子阱质谱联用的仪器系统,可进行原位成像分析。Haddad等^[105]^则是将简易敞开式声波喷雾电离(EASI或者DeSSI)源与TLC联用。EASI源的装置和操作方式与DESI类似,可用于大气压下固体表面样品的直接解吸电离,但是不需要使用高电压,而是使用声波喷雾产生气动液滴(如带酸的喷雾液滴),液滴中电荷分布的不平衡会导致带电液滴的产生。他们使用TLC-EASI-MS技术对药物、半极性化合物混合物和生物柴油等样品成分进行了表征^[106]^。而Mirabelli等^[107]^使用了和与DESI源相似的溶剂辅助解吸电离源系统进行表面样品的解吸和离子化,也无需加高电压,称为“DI”源,他们利用这种电离源与四极杆-离子阱质谱一起构成薄层色谱的检测系统。电离过程中,溶剂在高速喷雾挥发时可以形成过热雾,并使液滴带电,之后随着液滴的挥发,电荷逐渐集中而“爆炸”成为更细的带电微粒,而且这种电离不会因样品成分的相对分子质量大小而影响电荷分配^[108]^。他们结合SALDI技术使用这种电离源,比较几种具有不同理化特性和溶剂系统的支撑材料,如TLC板(C18、硅胶、氰基固定相)、聚四氟乙烯、玻璃、滤纸、金属(AnchorChip-MALDI板)等,结果表明硅胶TLC板作为脂肪酸样品(橄榄油、鱼油、鲑鱼和人血清)基底时对样品有较好的吸附-解吸和电离效果,其次是C18和氰基固定相材料;此外,pH和离子强度对电离效果也会有显著影响,这可能是因为这些因素影响了电荷转移的过程。

### 2.4 薄层色谱与大气压化学电离源质谱的联用

大气压化学电离(APCI)源也是常见的一种质谱电离源,将样品成分雾化后通过电晕针电离。Peng等^[109]^搭建了激光辅助解吸的TLC-APCI源系统,并结合了SALDI的方法分离分析磷脂类化合物(将石墨覆于TLC表面),实验结果表明这种方法能有效降低解吸-电离所消耗的激光能量^[110]^。Berkel研究组^[111,112]^则是在商品化Waters TQD质谱仪器基础上改装,使用加热探头对TLC板上的小分子目标成分进行热解吸ESI/APCI-MS分析。

解吸大气压化学电离(DAPCI)源使用加热的高速鞘层气体直接喷射样品表面,主要使用热解吸的方法使样品成分从吸附介质表面溢出,并通过电晕针在气相中电离。Winter等^[113]^搭建了一种大气压下DAPCI源,称为分子电离-解吸分析(MIDAS)源,可用于分析药物成分。它的原理与APCI源相似,但不同的是APCI源的电晕针位于喷雾附近,作用于雾化气和样品分子的混合物;而MIDAS源中电晕针设置在氮气加热管的出口处,用于给氮气充电,再用高温带电氮气来解吸并电离吸附在TLC板上的成分,之后目标成分带上电荷并进入飞行时间质谱进行分析。该装置设有样品传送带,可用于移动薄层板。

### 2.5 薄层色谱与即时直接分析质谱的联用

即时直接分析电离源质谱(DART-MS)起源于2005年左右,主要通过气体放电(通常以高压电晕针电离氦气)来生成等离子体激发态物质,激发态氦的原子流(可提供19.82 eV的能量)随着热气流并配合电场加速冲向样品,从而解吸并电离样品表面的物质;同时也可以激发大气中的水分子簇(水的一阶电离能为12.62 eV),并将质子转移到其他物质的分子上(如果使用氩气则不易激发水分子,因为其电离能小于水分子)。Gross等^[114]^对DART-MS的结构和原理进行了较为全面的综述。

在与TLC联用方面,Smith等^[115]^起初手持着TLC板,使薄层上4个谱带连续依次通过喷口,得到色谱图。Morlock研究组^[116⇓-118]^设计了不同的DART平台,并具体研究了TLC与DART源配合使用的具体参数,如位置、距离、角度等,提出了优化灵敏度的几个方法(1)提高气体温度,(2)减小薄层厚度,(3)使用基质辅助(例如在TLC板上喷撒甘油甲醇溶液以提高解吸和离子化效率)^[119]^。随后他们经过一系列改进(包括优化部件结构和布局、扫描通道、管路设计等方面,使仪器进一步结构紧凑化等)有效提高了仪器的性能^[120,121]^,可以分析低于ng级的样品。为了防止薄层板上邻近区域干扰而造成的“假阳性”信息,Morlock小组^[122]^继之前的研究后又着力于实现热气体的可视化,最终他们使用10% Ne(Ne的亚稳态能为16.61 eV)和He的混合气体实现了可视化的红色光效果,可以直接观察DART等离子体辉光,以便追踪薄层表面扫描过程中的亚稳气体分布,从而能够透过肉眼观察而直接对准目标谱带进行解吸和电离。

如前文所述,激光解吸能够有效提高目标成分的解吸-电离效率,刘虎威研究组将TLC技术与等离子体辅助多波长激光解吸电离(PAMDI)-MS相结合,使用多个波段的激光对TLC板的成分进行解吸,借助DART源作为离子阱质谱的离子源,可方便地分离和选择性鉴定相对分子质量较低的化合物^[123]^。

如前文所述,UTLC的固定相厚度小,一般被认为具有更高的灵敏度。然而,在Morlock研究组^[67,124]^的相关报道中,使用与HPTLC相同的离子源条件在(碳纳米管模板上沉积氮化硅制得的)UTLC表面扫描时,对比结果却显示HPTLC的信号强度优于UTLC,他们推测这可能是由固定相复杂的纹络结构和其晶体材料的热电性质造成的。因此,对于DART源来说,UTLC并非一定是最优的选择。

### 2.6 薄层色谱与流动大气压余辉电离源质谱的联用

Andrade等^[125,126]^开发了流动大气压余辉电离(FAPA)源,在聚四氟筒内引入He,并在该筒的一端设置钨针阴极,在钨针对面一端设置黄铜板阳极,两者间加载直流高电压引发He产生余辉放电,此气体从聚四氟筒中流出并电离固体表面上的样品分子,并进入质谱进行分析;之后Shelley等^[127]^改进了设计,使用接地的毛细管状喷口作为阳极,与钨针阴极组成电压对,有效地降低了背景噪声,并减少了样品成分的氧化。

Ceglowski等^[128]^结合了激光烧蚀和FAPA源的方法作为电离源,与四极杆-离子阱质谱联用,形成了TLC-MS系统。他们认为二极管激光器能够提供足够的能量用于解吸有机化合物,因而也无需像SLDI类似技术一样在样品点上添加石墨或其他物质,并使用这套装置分析了人参成分、吡唑衍生物、生物碱类天然化合物和药物片剂的活性成分。

Kuhlmann等^[129]^使用带有FAPA源的轨道离子阱质谱考察了TLC固定相对质谱信号的影响。实验中选定的样品成分有镇痛剂(对乙酰氨基酚)、生物碱(尼古丁和咖啡因)和类固醇(可的松),考察的固定相种类有正相硅胶、反相硅胶、腈基固定相、二醇基固定相和氨基固定相,建立了TLC-FAPA-MS来定量检测能量饮料中咖啡因的含量。此外,该研究中还对比了不同厚度的固定相对质谱信号的影响,值得注意的是,具有较大的粒径、更厚的固定相层更有利于得到较高的信号强度,而且极性较弱的固定相比高极性固定相有更好的质谱响应,如腈基固定相和反相固定相得到的信号丰度明显高于正相固定相。

### 2.7 薄层色谱与低温等离子体源质谱的联用

用于解吸、电离相关功能的低温等离子体仪器装置,一般会在流动的氦气环境下安装一对电极(其中一个电极被一个介电层覆盖),并施加交流电压,所产生的等离子体由激发态氦、离子、自由基和电子等物质组成。使用时等离子体流从发生器中射出,作用于固体表面样品,从而达到解吸和电离的目的。获得的质谱信息主要包含分子离子(如分析物的质子化分子和离子加合物)以及少量碎片离子。尽管这些等离子体被称为低温等离子体,它的温度其实并不低,在功率高于7 W的情况下,这些等离子体足以烧焦样品表面;为了最大限度地减少表面的可见热损伤,通常在<5 W的条件下运行电离源,使系统产生长度约为4~5 mm的细长等离子体射流;当小于3 mm时,目视检测时几乎观察不到表面上的任何损伤^[111]^。

Gong等^[130]^搭建的低温等离子体(LTP)源与DART相似,它由一根玻璃管(外径6.00 mm,内径4.00 mm)和它内部的一根钨棒(直径1.59 mm)组成,钨棒居中作为接地的内部电极,在玻璃管外围缠绕铜箔作为另一电极,高纯氦气作为放电气体,共同构成了离子源。他们使用这一离子源构建了激光解吸低温等离子体电离-质谱与薄层联用系统(TLC-LD-LTP-MS)。Garcia-Rojas等^[131]^搭建并使用类似系统,3 kV下引发He的低温等离子体进行电离,405 nm连续激光解吸,对薄层板上目标区域按点、线或面进行扫描分析,结合SALDI的方法还可以进一步提高解吸-电离效率。他们使用这套系统从咖啡、茶和可可制剂中分离甲基黄嘌呤,并进行了成像分析。

### 2.8 薄层色谱与其他电离源质谱的联用

簇诱导解吸-电离法(DINeC)使用中性SO_2_簇进行诱导解吸-电离,是一种软解吸-电离的方法。这种方式碎片较少,可用于多种物质(如肽、蛋白质、脂质和染料)的检测。分析过程中,中性SO_2_簇以较低的能量诱导成分进行解吸-电离,另一方面这些解吸出来的成分可溶解进簇中,而SO_2_分子可以从团簇中挥发出来,对这些样品分子进行冷却,从而有利于样品成分的稳定。Heep等^[132]^采用DINeC实现了TLC和离子阱质谱的联用,对从蛋黄中提取的不同磷脂进行分离和检测。

电感耦合等离子体质谱(ICP-MS)也可与TLC联用,而且常配备激光辅助解吸/烧蚀。TLC具有较快的分离速度和效率,成本低,多为一次性耗材,不涉及清洗色谱柱等操作,因此TLC-MS技术在石油行业一些成分分析中有独特优势^[10]^。例如使用薄层色谱与激光烧蚀-电感耦合等离子体-扇形场质谱联用(TLC-LA-ICP-SF-MS),可以对原油及其馏分(饱和油、芳烃、树脂和沥青质)中的砷、镍、钒、铁和硫等多种元素进行分析^[133⇓-135]^。而颜能等^[136]^使用以四极杆为质量分析器的MTLC(胶束薄层)-LA-ICP-MS仪器系统实现了不同粒径的纳米金、纳米银及离子的分离和检测。除此之外,TCL-ICP-MS也用于环境中分析,如Bednarik等^[137]^基于薄层色谱和二极管激光热蒸发-电感耦合等离子体质谱(DLTV-ICP-MS)相结合的方法测定了藻类和酵母中硒元素的含量。Kisomi等^[138]^制备了As(V)离子印迹聚合物作薄层固定相材料,用LA-ICP-MS对水溶液中无机砷进行分离和检测。

## 3 薄层展开的实时质谱监控技术

HPLC常与MS仪器直接联用进行实时监测,但是这种技术在TLC中却并不常见。在液相色谱-质谱联用的仪器系统中,有泵系统的支持,质谱电离源的主要功能之一是除溶剂;相比之下,除少数强制流薄层(如加压薄层和旋转薄层)外,其他TLC技术主要凭借毛细作用力驱动流动相,而没有外力支持,同时流动相的量也不大。另外,TLC的分离能力不及HPLC,这也是TLC-MS应用不及HPLC-MS的主要原因。总体来看,实时在线检测的TLC-MS可以分为强制流薄层色谱-质谱联用和常规薄层色谱-质谱联用,这里说的强制流薄层色谱是以毛细作用力和其他辅助作用力共同驱动展开剂流动的TLC方法,而常规薄层色谱法则是指仅凭借毛细作用力为驱动力的TLC方法。

在强制流TLC方面,旋转薄层的情况与HPLC有相似之处,因此Berkel等^[139]^曾使用简单的管路设计即实现了旋转制备薄层色谱系统与质谱联用。聂宗秀研究组^[58]^开发了实时在线TLC-MS分析系统,由强制流TLC系统(流动注射法供给TLC展开液)、Ar辉光放电-基质辅助红外解吸电离(GD-MAIRDI)源,以及线性离子阱质谱系统组成,使用过程中脉冲光源对薄层分离的影响较小,可以实现在线的连续检测。Morlock研究组^[140]^则使用加压薄层与四极杆-轨道离子阱质谱联用,并结合生物自显色的方法分析了丹参中的聚乙酸烯等活性成分。

而常规TLC方面,Shiea研究组^[52]^设计了TLC-ESI-MS接口装置,使用聚四氟乙烯为基板,上面刻有固定相通道,内置固定相;通道起始端连接圆槽状流动相池,通道接近末端处可以设圆槽状流动相补充池,流动相池或者补充池与高压电源相连;通道末端为尖头金属或者光纤,用于产生电喷雾,对面安装毛细管质谱接口,可直接收集和传导样品成分离子。此类系统还可以在聚四氟板上增加通道数,配备用于TLC板移动的平台,便于提高检测通量^[141,142]^。

## 4 效应导向薄层色谱与质谱的联用

效应导向分析(EDA)是指将化学分析与生物测试相结合,用于识别样品中有活性或者毒性的成分(如天然药物、代谢产物、副产物、工艺杂质/污染物、降解产物、掺假药物和残留物等)的技术,在TLC中的应用常被称为薄层色谱-生物自显影技术,例如自由基清除活性、酶抑制剂活性、微生物抑制活性化合物的检测和物质筛选,或者免疫检测等。如果将生物导向与薄层-质谱分析相结合,则可以有效提高活性化合物筛选的工作效率。近年来,这些应用一般体现于食品和生物制药领域,在TLC-MS联用方面多采用接口仪器装置,表1列举了部分应用实例(表中示例均为直接在薄层板上进行生物自显影类检测方法,而非使用接口设备技术将成分转移出来进行生物活性实验)。

**表1:** TLC-EDA-MS用于分离和分析的示例总结

Field	Solid phase	Samples	Ref.
Food	NP	anthocyanin content in applesauce, feed, fruit juice and wine	[143]
	NH_2_	pesticide residues in apple, red grape, cucumber and other samples	[43]
	NH_2_	multiple antibiotic residues in animal samples such as milk	[144]
	NP	stability of saponins in cooking	[145]
	NP	cold pressed hemp, flax and rapeseed oil	[146]
	NP	aflatoxin deoxynivalenol in wheat	[31]
	NP	cinnamon powder and food containing cinnamon	[32]
	NP	coffee bean fingerprint	[33]
	NP	monomeric and polymerized anthocyanins	[147]
	NP/RP/NH_2_	flavonoids and antioxidants in fruits and finch leaves	[34]
Biology & medicine	NP	triterpenoids and phytosterols	[148]
	NP	medicinal mushroom	[149]
	NP	neutral sphingolipids	[150]
	NP	monoglycerides in biodiesel	[151]
	NP	active substances in Solidago virgaurea L.	[152]
	NH_2_	ergot alkaloids in rye flour	[153]
	NP	cholinesterase (AChE) inhibitors in plant extracts	[154]
	NP	AChE inhibitors in plant extracts (77 plant extracts)	[32]
	NP	lichen metabolites	[36]
	NP	neutral lipids and sphingolipids	[40]
	NP	sandalwood root extract	[37]
	NP	phospholipids related to membrane proteins	[41]
	NP	cuticular lipids	[42]
	NP	plant extracts from three different years	[38]
	NP	the genotoxicity of metformin	[35]
	NP	drug composition in blood	[155]
	NP	phenolic acids and terpenoids in Flos Lonicerae	[39]

EDA: effect directed analysis; NP: normal phase; NH_2_: amino based solid phase; RP: reversed-phase.

## 5 总结与展望

薄层色谱在化学合成、食品、药品、刑事侦查、消防与安全检测领域都有着重要的地位;随着生物自显影的应用,薄层色谱与复杂基质中活性物质基础之间的关联得以加强,这无论对新药物质的发现还是临床检测来说都有很好的意义。质谱技术的引入无疑拓展了TLC的检测能力,尤其是定性能力;另一方面,对于TLC原位检测应用来说,结合MS扫描成像的应用和TLC高通量的特点,也将有利于提高质谱的检测效率,丰富数据信息。

如前文所述,薄层与质谱的联用大致可分为离线联用、原位检测和实时监测,三者各有特点。(1)离线联用的灵活性最好,可以通过接口装置有针对性地进行目标谱带的提取和检测,而且容易获得商品化仪器的支持,但不足之处是每次仅能处理一个目标谱带。(2)原位分析是在TLC分离结束后进行MS检测,具有TLC高通量的特点,通过MS扫描可大大提高分析效率,但目前可支持商品化仪器较少(如BrukerDaltonics的MALDI适配器,IonSense的DART源),而且价格昂贵;另外,本文引用的文献研究中涉及的质谱分离器种类多样,包括双聚集、四极杆、离子阱(线性离子阱或轨道离子阱)、飞行时间等,这说明质谱分析器的种类并非TLC-MS联用的限制因素;因此,“即插即用”型的离子源设计与商品化仍是限制TLC-MS发展的主要因素。(3)实时监测法可获得TLC的在线分离信息,但目前这种做法仅和强制流薄层技术联用时比较容易实现。如能实现常规TLC上的应用,并引入灵活的动态扫描模式,即可实现高通量检测,这将是个很好的技术优势,可以弥补HPLC-MS技术的不足。

对于TLC固定相来说,一般较薄的固定相厚度有利于样品成分的解吸,因此UTLC板在灵敏度方面更具优势;但这并不是绝对的,具体情况也需要考虑到TLC表面结构和性质,正如前文中提到的DART源中出现了HPTLC的响应优于UTLC的情况;有意思的是,FAPA源在应用中也出现了对粒度较大、厚度较厚的固定相有更高解吸效率的情况,考虑到这两种电离源都是基于等离子体技术的应用,因此等离子体类电离源技术的特点可能仍需进一步探索。另外,比较遗憾的是尽管与TLC联用的质谱离子源种类丰富,但不同离子源在TLC-MS联用中的对比研究却并不多,相信这些差别不仅来源于电离源,同时还可能与TLC表面性质、基质作用、解吸过程,以及样品成分类别等方面有关。

总之,TLC-MS已经引起了人们的关注,尤其是薄层-生物自显影等技术的广泛使用,更突显了质谱与薄层色谱结合的迫切性。如何在现有仪器基础上进行“即插即用”型部件的设计与商品化是目前推广这一技术的主要瓶颈,值得关注;同时兼备灵活扫描功能、高通量和实时监测功能的TLC-MS技术也很令人期待。另外,各原位检测TLC-MS方法的解吸-电离方式各有特点,它们之间的对比也是有待于进一步讨论的应用问题。相信不久的将来,这些问题会一一解决,而TLC-MS技术也会有更丰富的应用。
